# Mesoporous Silica Nanoparticles and Mesoporous Bioactive Glasses for Wound Management: From Skin Regeneration to Cancer Therapy

**DOI:** 10.3390/ma14123337

**Published:** 2021-06-17

**Authors:** Sara Hooshmand, Sahar Mollazadeh, Negar Akrami, Mehrnoosh Ghanad, Ahmed El-Fiqi, Francesco Baino, Simin Nazarnezhad, Saeid Kargozar

**Affiliations:** 1 Pharmacological Research Center of Medicinal Plants, Mashhad University of Medical Sciences, Mashhad 917794-8564, Iran; s_hooshmand@yahoo.com; 2 Department of Pharmacology, Faculty of Medicine, Mashhad University of Medical Sciences, Mashhad 917794-8564, Iran; 3 Department of Materials Engineering, Faculty of Engineering, Ferdowsi University of Mashhad (FUM), Azadi Sq., Mashhad 917794-8564, Iran; mollazadeh.b@um.ac.ir (S.M.); negari.akramii@gmail.com (N.A.); mehrnooshghanad@gmail.com (M.G.); 4 Glass Research Department, National Research Centre, Cairo 12622, Egypt; dr.ahmed.elfiqi@gmail.com; 5 Institute of Materials Physics and Engineering, Applied Science and Technology Department, Politecnico di Torino, Corso Duca degli Abruzzi 24, 10129 Torino, Italy; 6 Tissue Engineering Research Group (TERG), Department of Anatomy and Cell Biology, School of Medicine, Mashhad University of Medical Sciences, Mashhad 917794-8564, Iran; smn.nazarnezhad@yahoo.com

**Keywords:** mesoporous silica nanoparticles, mesoporous bioactive glass, angiogenesis, skin regeneration, cancer therapy, drug release, tissue engineering

## Abstract

Exploring new therapies for managing skin wounds is under progress and, in this regard, mesoporous silica nanoparticles (MSNs) and mesoporous bioactive glasses (MBGs) offer great opportunities in treating acute, chronic, and malignant wounds. In general, therapeutic effectiveness of both MSNs and MBGs in different formulations (fine powder, fibers, composites etc.) has been proved over all the four stages of normal wound healing including hemostasis, inflammation, proliferation, and remodeling. The main merits of these porous substances can be summarized as their excellent biocompatibility and the ability of loading and delivering a wide range of both hydrophobic and hydrophilic bioactive molecules and chemicals. In addition, doping with inorganic elements (e.g., Cu, Ga, and Ta) into MSNs and MBGs structure is a feasible and practical approach to prepare customized materials for improved skin regeneration. Nowadays, MSNs and MBGs could be utilized in the concept of targeted therapy of skin malignancies (e.g., melanoma) by grafting of specific ligands. Since potential effects of various parameters including the chemical composition, particle size/morphology, textural properties, and surface chemistry should be comprehensively determined via cellular in vitro and in vivo assays, it seems still too early to draw a conclusion on ultimate efficacy of MSNs and MBGs in skin regeneration. In this regard, there are some concerns over the final fate of MSNs and MBGs in the wound site plus optimal dosages for achieving the best outcomes that deserve careful investigation in the future.

## 1. Introduction

Skin is the human body’s largest organ, which acts as the outer protective system of the body. This organ is composed of three distinct multi-tissue layers, including the epidermis, dermis, and hypodermis. Each layer has its unique structure and function, resulting in mechanical and biological differences among different parts of the skin across the body. It has been well documented that the skin and its compartments are able to communicate with other tissues (e.g., muscles) and to make hemostasis via the production of different cytokines, neurotransmitters, hormones, and their relevant receptors. In addition, this organ is a vast reservoir of various stem cells that participate in the rebuilding process of damaged regions. Accordingly, the skin supports an individual’s survival via exerting different functions, including the protection against harmful physical (e.g., ultraviolet radiation), chemical (e.g., toxins), and biological (e.g., microbes) agents, the prevention of water loss, and the regulation of body temperature [1]. Due to its extension, the skin is susceptible to a variety of damages and injuries ranging from a simple scratch to life-threatening cancers. Since skin lesions may adversely affect life quality, numerous attempts have been made to generate applicable remedies in regenerating appropriate tissue substitutes in the laboratory. On this matter, outstanding progress has been made during the last decade, and emerging technologies are being entered the market [2,3,4]. Among them, applying mesoporous nanoparticles, either alone or in combination with other biomaterials such as soft polymers, has shown great promise due to their excellent physicochemical and biological properties.

Mesoporous silica nanoparticles (MSNs) are nano-sized silica (SiO_2_) particles that possess pores with the size of 2 to 50 nm in their structure. These materials have a long successful history in medicine, especially those applied in targeted drug delivery and tissue engineering [5]. Taking advantage of high specific surface area, mesoporous structure, tunable size/shape, good biocompatibility, and stable aqueous dispersion, MSNs have attracted huge interest for diagnostic and therapeutic medicinal purposes in recent decades [6,7]. Moreover, the surface of MSNs provides great opportunities for grafting functional groups and diverse therapeutic macromolecules, making them highly versatile substances in biomedical applications [8]. Through some innovative approaches like capping strategies, it is feasible to provide MSNs with smart properties for intelligent drug delivery so that they can respond to various stimuli (e.g., pH changes) and unload the cargo for targeted therapeutic applications [9]. Focusing on wound healing, in vitro and in vivo experimental data support the positive role of MSNs in skin regeneration [10,11].

If additional oxides (e.g., CaO, P_2_O_5,_ or small amounts of metallic oxides used as dopants) are added to pure silica, then mesoporous bioactive glasses (MBGs) are obtained. They are a well-known subset of the BGs family with appealing multifunctional properties for a range of biomedical applications (drug delivery, tissue regeneration, and cancer therapy) [12,13]. In principle, both MSNs and MBGs can be synthesized by similar “wet methods,” like sol–gel process, are amorphous materials, exhibit ultra-high specific surface area (up to 1000 m^2^/g) and tunable pore size in the meso-range depending on the synthesis parameters (e.g., type of surfactant used, pH, temperature, etc.) The main differences between MBGs and MSNs are associated with the bioactivity, biodegradability, and bioactive ion release capability of MBGs, which initiate after their immersion in a biological fluid (e.g., plasma); on the contrary, MSNs are almost non-bioactive and undergo very slow dissolution upon contact with aqueous media. The ordered nanoscale pore structure (pore size of 2–50 nm) of MBGs plays a critical role in promoting their fast apatite-forming ability. Having the mesoporous structure, MBGs are also being applied for loading and delivering different drugs (e.g., antibiotics) and bioactive macromolecules (e.g., growth factors). The possibility of MBGs to serve as multifunctional biomaterials is another advantage in tissue engineering and regenerative medicine [14,15]. The main application of MBGs is to treat hard tissues; this is due to the inorganic nature and mechanical rigidity of all bioactive ceramics and glasses, which exhibit physical characteristics closer to calcified tissues like bone. Furthermore, they exhibit beneficial properties for improving bone tissue healing: for example, silica-based MBGs can make a strong bond with host bone tissue and interact with bone-forming cells (osteoblasts), leading to up-regulating the expression of genes and proteins that govern the osteogenesis process [16]. However, recent evidence indicates MBGs suitability in managing skin wounds as well. On this matter, the reported studies have been full of hopes and promise [17]; however, more research should be carried out to determine all the pros and cons in the way of extensive usage of MBGs in skin wound healing. For example, their optimal formulation and dosages, as well as final fate in injured sites, indeed deserve careful investigation in the future. As a step forward, the evaluation and comprehensive understanding of different parameters of MBGs, including their chemical composition, particle size/morphology, bioactivity, biodegradability, ion release, textural properties, and surface chemistry, may be helpful in designing more suitable substances for skin wound repair and regeneration.

In the present study, we, for the first time, review the importance of MSNs and MBGs in treating and managing different skin wounds and malignancies to urge on considering these promising substances in the next-generation therapies. Based on the reported data in the literature, the advantages and disadvantages of these materials are comprehensively described and critically discussed to open new rooms for researchers and scientists in this emerging area of science.

## 2. Wound Healing Process

Generally, skin wound healing comprises four overlapping phases with a well-orchestrated interaction of specialized cells, bioactive molecules, and extracellular matrix (ECM) components. In the first phase, the so-called hemostasis, the formed fibrin clot provides a temporary scaffold for cellular attachment and migration [18]. In addition, platelets produce pro-inflammatory cytokines (e.g., PDGF and TGF-β) that contribute to the inflammatory phase. The inflammatory phase is the second phase in which neutrophils are infiltrated and support reinforcing immune response through releasing TNF-α, IL-1β, and IL-6. Furthermore, monocytes migrate to the wound bed and differentiate into macrophages that serve as phagocytes and release bioactive molecules including IL-1, PDGF, TGF-α, TGF-β, FGF, IGF-1, and VEGF [19]. The third phase, i.e., proliferation, is remarked by re-epithelialization, angiogenesis, and granulation tissue formation. In the last phase, i.e., remodeling, the residing collagen type Ⅲ in granulation tissue is replaced by bundles of collagen type Ⅰ that result in scar tissue formation and subsequently enhance the tensile strength of ECM [20].

Etiologically, the skin wounds could be classified as acute, chronic, or malignant wounds. In general, an ordered and normal wound healing process happens after acute wounds [21]. On the contrary, chronic wounds are characterized by prolonged inflammatory phase [22], sustained infection [23], and the generation of drug-resistance microbial biofilms [24], leading to an impaired wound healing cascade. In the case of malignant lesions, the edges of wounds are constantly expanded because of the cell migration inward and outward of the wounds, which results in tumor proliferation as well as its invasion into the adjacent tissues [25].

It is well-known that successful and efficient wound healing requires improved angiogenesis, re-epithelialization, and less fibrous and scar tissue formation. Hence, numerous studies have been focused on developing innovative wound substitutes and dressings, which are summarized in the next sections.

## 3. Current Therapies in Managing Wounds and the Potential of Mesoporous Materials

Traditional wound dressings, including gauze, biofilms, ointments, and creams, serve as a protective barrier against the external environment and, hence, infections. Despite their low cost and ease of use, traditional dressings may cause necrosis/ischemia and need to be repeatedly replaced. In contrast, advanced dressings play an active role in treating more serious damages [26]. These types of remedies include biocompatible and biodegradable materials, which are made of both natural and synthetic substances capable of providing accelerated tissue repair and regeneration [27]. An ideal wound dressing should have a series of suitable physico-chemical, mechanical, and biological properties, including the proper ability to absorb water, the maintenance of a moist environment for the wound bed, low cytotoxicity and immunogenicity, antibacterial properties, gas permeability, and the ability to absorb wound exudate. All the mentioned criteria could contribute to a better and faster wound healing process [28]. In this concept, experimental data highlight that the use of three-dimensional (3D) structures (scaffolds) is considered an emerging strategy due to the possibility of delivering various bioactive molecules and cells into the injured regions. Hydrogels, electrospun nanofibers, and 3D bio-printed constructs are among the most widely used scaffolds for promoting the wound healing process [29,30,31,32]. These scaffolds could resemble the native ECM architecture as well as provide a substrate for incorporating cytokines, growth factors, phytochemicals, and other bioactive molecules. For example, an electrospun nanofibrous mat composed of poly(caprolactone) (PCL) and poly(ethylene glycol) (PEG) loaded with 0.5 wt% curcumin (with respect to PCL) showed an enhanced rate of wound closure (99%) on day 10 compared to only PCL nanofibrous mat (59%) in a mouse model [33]. In addition, hard ceramics (e.g., bioactive glasses) have been recently introduced as promising materials in soft tissue engineering as they could positively affect the wound healing process [34].

**Figure 1:**
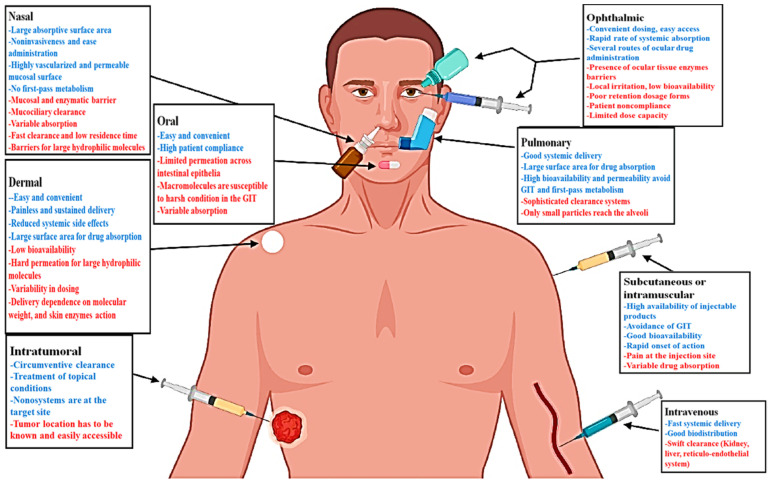
Schematic illustration of the routes generally used for MSN administration, including their main advantages (blue) and challenges (red). Reproduced with permission from ref [41]. Copyright 2021 Elsevier.

MSNs have a long successful history in treating and managing different damages and injuries related to both hard to soft tissues and organs. They exhibit excellent properties in the case of the medical setting; for example, it is possible to administrate bioactive molecule-loaded MSNs by several routes (Figure 1). Moreover, these versatile materials could be easily added to polymeric matrices and prepare nanocomposites capable of filling tissue defects. Over time, MSNs were found as potential substances in managing soft tissue lesions, including skin infections [35]. They could also serve as appropriate platforms for transdermal delivery of a broad range of anticancer agents (e.g., chemicals, drugs, and small interfering RNAs), which may facilitate skin cancer treatment [36,37,38]. Incorporating therapeutic ions (e.g., Zn^2+^, Eu^3+^, and Cu^2+^) into the MSNs has become a highly promising approach for eliciting specific biological responses in damaged sites, including antibacterial, anti-inflammatory, and proangiogenic activities [11,39,40].

More recently, the usability of hard ceramics (e.g., bioactive glasses and glass-ceramics) in soft tissue engineering have been comprehensively reviewed by Kargozar et al. [34]. These substances have shown great promise in managing both acute and chronic wounds, and their emerging applications in treating skin malignancies are still under evaluation [17]. Bioactive glasses were previously proven as suitable additives for promoting different stages of skin wounds [42,43]. Up to now, various formulations of bioactive glasses, including silicate-, phosphate-, as well as borate- and borosilicate-based glasses, have been investigated in treating skin injuries with promising outcomes [44,45,46]. It should be pointed out that researchers often deliberately incorporated a range of metallic elements (e.g., Cu and Co) into the glass structure as dopants to provide glasses with specific biological features (e.g., improved angiogenesis) that are useful for obtaining an accelerated wound healing [47,48]. Still, there are huge numbers of questions that should be addressed before their approval in clinical studies, including their early and late effects in the body, optimal dosages, and preferable administration routes. A specific type of glasses, i.e., mesoporous bioactive glasses (MBGs), offer great opportunities in tissue engineering with respect to their capability of drug loading and delivery [12,13,16,49]. Prior studies have confirmed MBG suitability in soft tissue repair and regeneration; they could promote hemostasis, angiogenesis, epithelial cell migration, and fibroblast cell proliferation. In order to meet basic needs in the wound healing process, the preparation of composites made of MBGs and different biopolymers (natural and synthetic) have gained much attention [50,51]. In addition, different modifications, including surface functionalization, could be applied to MBGs to make them suitable materials for targeted therapeutic approaches [52].

## 4. MSNs: Classification, Preparation Methods, and Biocompatibility

MSNs with a pore size of 2–50 nm in diameter were first reported as molecular sieves in the early 1990s due to their inherent structural characteristics [53]. Nowadays, MSNs are being applied in a broad range of applications, including biosensors, catalysis, energy storage, and—most importantly—drug delivery due to their large internal surface area leading to unique external surface reactivity [54]. MSNs used for drug delivery are classified into four types of (I) traditional MSNs, including Mobile Crystalline Materials (MCMs) [55], Santa Barbara Amorphous system (SBA family) [56], Korea Advanced Institute of Science and Technology system [57], and Technische Universiteit Delft (TUD) system [58]; (II) hollow MSNs (HMSNs), (III) lipid bilayer-coated MSNs (LB-MSNs) [59]; and (IV) modified MSNs having to target moieties, polymers, and organic functional groups to control the adsorption and release of the drugs in targeted drug delivery [60].

In general, MSNs can be synthesized via four different methods, including the template-directed method [61], the sol–gel method [62], the microwave-assisted technique [63], and the chemical etching technique [64]. The morphology of MSNs, particularly the size and shape, could considerably affect theirs in vitro interactions with cells and cellular components as well as their in vivo performance in the living systems. The pore size of MSNs is another crucial factor impacting their biological performance and should be correlated, for example, to the size of the drug molecule to host. In addition, the surface chemistry of MSNs was confirmed as a remarkable parameter playing a role in their biocompatibility, drug release behavior, specific targeting ability as well as outcomes of their interactions with stem cells or scaffolds. MSNs could be easily functionalized with a group of diverse ligands thanks to their high density of surface silanol groups, making it easier to achieve more favorable surface properties needed for tissue engineering strategies. The surface of mesoporous silica can be engineered for imparting special extra-functionalities to the material. For example, Izquierdo-Barba et al. [65] functionalized SBA-15 particles with amino (–NH_2_) groups, carboxylic groups (–COOH), or both to compare the effect on the adhesion of *Escherichia coli*. It was reported that bacteria could colonize the surface when only amino or carboxyl groups were present, while the amino-carboxyl bi-functionalization showed clear antibacterial properties. Surface functionalization of the silica walls may also be necessary to modulate the drug uptake and release. Some drugs exhibit a remarkable hydrophobic nature and, thus, are not prone to enter the pores of hydrophilic mesoporous silica. The surface functionalization with hydrophobic functional groups is a good strategy to overcome this limitation, promoting the incorporation of different hydrophobic drugs. This strategy is also useful to delay the release kinetics of certain drugs from the mesopores to the biological aqueous fluids due to the decrease in the wettability degree of the material surface. In these cases, higher drug loads and slower release kinetics can be achieved if the mesoporous silica walls are covalently functionalized with amino groups. In other situations, a strong interaction such as direct covalent bonding between the silicate biomaterial and the therapeutic biomolecule (e.g., growth factor, anticancer agent) is advisable to allow a quite prolonged effect in the long term. A detailed picture of these functionalization strategies can be found elsewhere [66].

Regarding in vivo applications, particular attention has been given to the toxicity and biocompatibility of MSNs to determine their genotoxicity, cytotoxicity, and blood and tissue compatibility. It is reported that endocytosis pathways are predominant translocation mechanisms for MSN internalization into cells [67,68]. Although the genotoxicity of MSNs has not been extensively investigated, prior results indicate that MSNs might cause genotoxicity to normal human cells to some extent, and caution should be taken regarding their extensive use [69,70]. All effective factors determining genotoxic outcomes of MSNs could be classified into two major groups: (I) MSN physicochemical properties (e.g., the size, charge, agglomeration state, porosity, surface properties, etc.) and (II) treatment conditions (e.g., applied dosage, exposure time, cell type, and animal model) as well as single or consecutive treatments [71]. Due to significant cytotoxicity of high-dose MSNs (>200 μg/mL) as well as the negligible cytotoxicity of low dosage (<50 μg/mL), it is strongly recommended to complete removal of toxic surfactants from the pores of MSNs by extraction or calcination prior to drug loading applications [72]. Recently published reports indicate that the incorporation of specific elements (e.g., strontium) into MSNs significantly enhances cytocompatibility [73]. In cases of blood compatibility, parameters including the concentration and size of MSNs may directly affect the final outcomes; long-range ordered porous structures may cause red blood cell membrane damage [74]. Interestingly, the presence of silanol groups on the cell-contactable surface of MSNs is proposed as one of the main reasons behind their hemolytic activity [74]: therefore, caution should be suggested when MSNs are used in direct contact with blood. In contrast, it has been reported that MSNs possess immunomodulatory effects through the inhibition of the Wnt5A/Ca^2+^ pathway and the activation of autophagy upon uptake by macrophages [75]. However, when tested in zebrafish, MSNs have shown to induce no obvious histological or pathological abnormalities [76].

## 5. Multiple Roles of MSNs in Skin Wound Healing

As mentioned above, MSNs could act as promising carriers for loading and delivery of a broad range of chemicals, small bioactive molecules, and drugs due to their large pore volume and high surface area [77]. Spatially and temporally controlled release of drugs is achievable by taking advantage of the flexibility and tunability of MSN morphology. For instance, the pore size of MSNs can directly affect cargo release profile and subsequent cellular responses as well as a tissue repair process; in fact, the larger pore sizes lead to faster drug release rates and vice versa [78]. In order to develop more precise drug delivery systems, MSNs can be functionalized by various agents, including temperature- [79], light- [80], pH- [81], magnet- [82], enzymes- [83], and redox-responsive mediators [84]. Experimental studies have confirmed the suitability of drug-loaded MSNs in accelerating the wound healing process [85,86].

**Figure 2:**
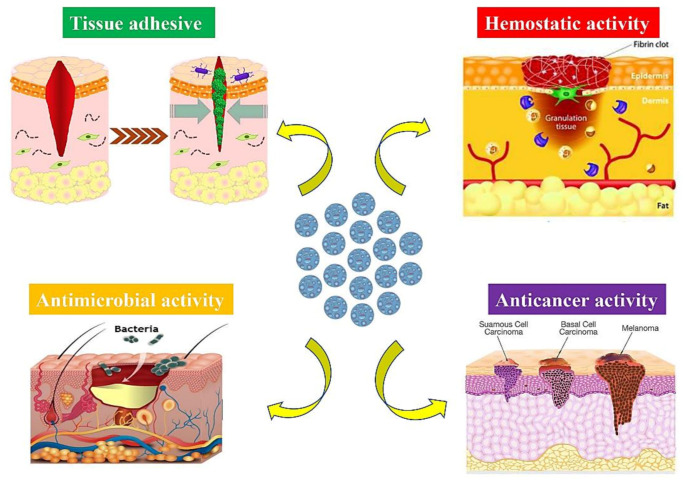
Schematic illustration displaying possible capacity of mesoporous silica nanoparticles (MSNs) (the blue circles in the middle) for managing different skin diseases and disorders.

MSNs are already identified as suitable platforms for topical drug delivery; formulated drugs are directly applied to be efficiently absorbed on the skin. Topical drug delivery is generally applied to overcome the limitations of conventional administration methods (e.g., oral and parenteral), including systemic side effects or needle phobia. Topically delivered agents/drugs with therapeutic concentrations cause less organ toxicity due to their accumulation within the target site of application, which maintains low serum levels [87]. In topical drug delivery, formulated drugs are directly applied to be efficiently absorbed on the skin. In order to reach systemic circulation, the drug must consecutively permeate the skin layers. It is worth underlining that three layers of skin along with relevant appendages are actively engaged in the topical drug delivery, including the epidermis, the dermis, and the hypodermis, plus hair follicles, sweat glands, and nails [88]. After being released, the drug penetrates into the stratum corneum (the outermost layer of the epidermis) followed by passing to the more aqueous layer of the epidermis and is finally absorbed via the dermis capillaries. As the key barrier to drug penetration, the stratum corneum signifies a cornified cell envelope, which is made of low-hydrated and high-density layers of stretched flat corneocytes with a densely packed lipid/protein polymeric structure below it. Despite being more permeable, the other layers and appendages only provide some target drug delivery sites [89]. The use of silica nanoparticles as carriers has been widely suggested to locally administrate the agents/drugs and store them into the skin appendages in order to facilitate crossing the skin multilayer barrier [90,91]. After the chemical agents/drugs overcome constituent layers of skin, they can be absorbed by the skin through sweat glands, hair follicles, or via the intracellular or intercellular routes by partitioning into the lipid matrix [92]. In the case of the hair follicle route, it has been verified that it forms a substantial reservoir for topically functional molecules [93] and nanoparticles [94,95] as a result of its functional location, which mediates the diffusion across the capillary walls of the stored molecules to the surrounding areas. MSNs have been previously utilized for enhancing the strength and adhesiveness of hydrogels to skin for transdermal drug delivery [96]. In fact, the cohesive property of the constructs can be improved as a consequence of molecular interactions between MSNs and polymer chains. Still, potential toxicological concerns about the transdermal delivery of nanomaterials are still an issue. The interactions of nanoparticles (NPs) with human skin surfaces have been shown to be influenced by shape, size, z-potential, surface charge, and tendency to aggregate as critical factors affecting the performance of the nanocarrier [95,97,98]. Regarding the significance of size, for instance, it has been confirmed that silica NPs with an average size of <25 nm can penetrate but not permeate the skin, while only NPs below 1 nm have the ability to generally permeate the intact skin. Moreover, after one or five days of topical application, NPs with higher diameters (55 ± 6 nm) could not pass the perturbed or normal mouse skin [95]. In the following sections, the therapeutic possibilities of MSNs are evaluated in the case of skin wound healing, including their hemostatic, antimicrobial, tissue adhesive, and anticancer activities (Figure 2).

### 5.1. Hemostatic Wound Care

Uncontrolled bleeding is stated as the cause of 15–25% of trauma deaths and over 50% of battlefield casualties [99,100]. An ideal hemostatic material should have the capability to form blood clots rapidly with sufficient stability for accelerating wound healing. In addition, biodegradability, biocompatibility, bactericidal/bacteriostatic activities, and low cost-benefit ratio are additional features for any materials used for managing hemostasis. Mesoporous nanosilica derivatives have shown the capability of immediately and efficiently controlling the bleeding; their morphological characteristics, textural properties (i.e., surface area, pore size, and total porosity), and surface chemistry are the main determinants governing their hemostatic functions [101,102,103]. As an illustration, Li et al. added curcumin (4 wt%)-loaded MSNs (CCM-MSNs) into poly(vinyl pyrrolidone) (PVP) nanofiber mats to fabricate a hemostatic and antibacterial substitute. After being contacted with blood, this hybrid system rapidly gelified and could activate the clotting system in vivo to stop the wound bleeding [104]. In another study, tannic acid-loaded MSNs were designed for overcoming the challenges of massive bleeding and bacterial wound infection [105]. This construct could efficiently promote protein adhesion and activate signaling pathways involved in the coagulation cascades, resulting in a reduced in vitro and in vivo hemostatic time (up to 65%) along with lower blood loss, improved antibacterial activity, and excellent cell viability.

### 5.2. Antibacterial and Antifungal Strategies

MSNs are continuously being introduced as suitable platforms for treating clinical antibiotic-resistant bacterial infections. A wide range of antimicrobial agents (e.g., peptides, antibiotics, and herbal extracts) were successfully incorporated into either bare or functionalized MSNs and utilized for managing wound infections [106,107,108]. For instance, silver-decorated MSNs were coated onto single-wall carbon nanotubes (SWCNTs) to improve their dispersibility and, consequently, increase their contact area with bacterial cell walls [109]. Indeed, the mesoporous structure of MSN layers enhanced the antibacterial activity by acting as micro-reactors to improve uniform distribution and control the small size of Ag nanoparticles. This nanosystem, via a fast release of Ag^+^ ions, showed much stronger in vitro antibacterial performance against multi-drug-resistant bacteria *Staphylococcus aureus* (*S. aureus*) and *Escherichia coli* (*E. coli*) as compared to commercial silver nanoparticles. In vivo studies on a rat model of skin infection also proved remarkable biocompatibility as well as outstanding abilities of this system in promoting wound healing and bacterial clearance. For utilizing MSNs in synergistic antibacterial therapy of multidrug-resistant bacteria, silver–bismuth-containing MSNs (Ag-Bi@SiO_2_ NPs) were developed and assessed in vitro and in vivo [35]. The hyperthermia originating from Bi nanoparticles could disrupt bacteria cell integrity as well as accelerate the Ag^+^ ions release from MSNs, resulting in an excellent antibacterial activity against methicillin-resistant *S. aureus* (MRSA). In addition, 100 µg mL^−1^ of Ag-Bi@SiO_2_ NPs could effectively eliminate mature MRSA biofilm and cause a 69.5% decrease in the biomass under laser irradiation, exhibiting a 30.8% improvement compared to those receiving no laser treatment. More importantly, in vivo data indicated that the Ag-Bi@SiO_2_ NP-based antibacterial platform kills nearly 95.4% of bacteria in the abscess and accelerates the abscess ablation. Concerning antifungal applications, it has been previously reported that mesoporous silica-coated Ag nanoparticle-based hybrid photosensitizers could exhibit an appropriate activation against dermatophyte Trichophyton rubrum [110]. In addition, prior experiments have shown that encapsulation and delivery of antifungal agents (e.g., herbal extracts and chemicals) using MSNs can significantly enhance their antifungal effects [111,112]. On this matter, MSNs, with an average pore size and diameter of 2 and 500 nm, respectively, were synthesized and modified with a photosensitizer (i.e., Rose Bengal (RB)) to be used in photodynamic therapy (PDT) for treating *C. Albicans* biofilms [113]. By emitting light, MSNs-RB exhibited high antimicrobial action against *C. Albicans* planktonic cells (reduction up to 88.7%) and biofilms (reduction up to 79.7%) as compared to RB-free MSNs. The production of high levels of reactive oxygen species (ROS), protein leakage (from the cytoplasm of *C. Albicans* cells), DNA damage, and lipid peroxidation were recorded as therapeutic effects of this nanosystem against infections caused by antifungal drug-resistant and biofilm-forming strains. For overcoming the low solubility of Econazole (ECO), an antifungal widely applied agent for topical infection treatment, MSNs modified with aminopropyl groups were developed to be used as a delivery system [114]. The reported data indicated that ECO-loaded MSNs had a greater antifungal activity compared to the standard ECO cream in rabbits, suggesting the usefulness of MSNs for the treatment of the infections caused by fungi (e.g., *C. Albicans*) without any significant signs of induced skin irritation.

### 5.3. MSNs as Tissue Adhesives for Wound Closure

**Figure 3:**
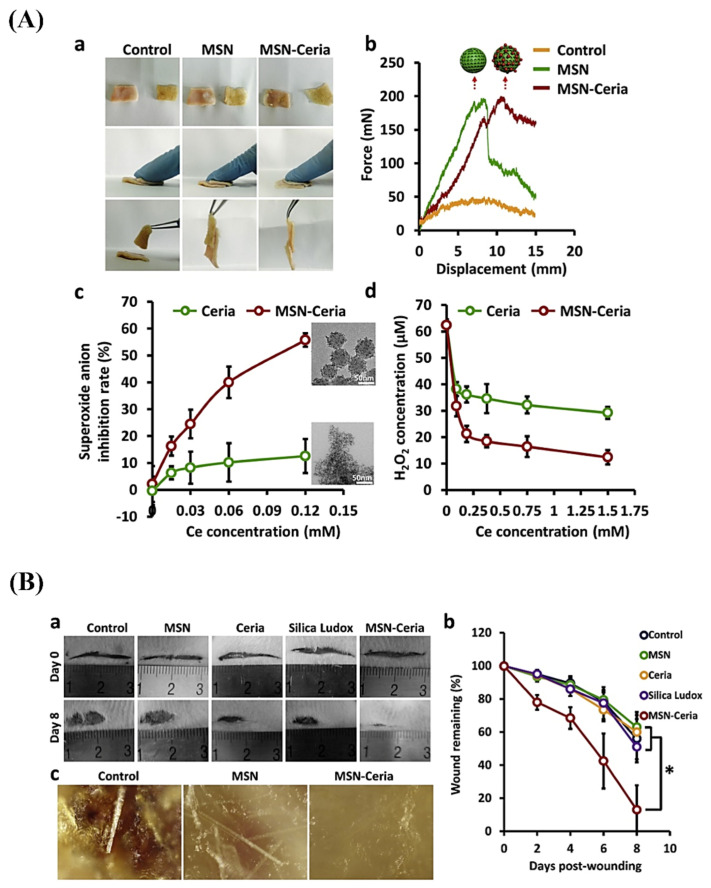
(**A**) Aqueous solutions of MSN and MSN-ceria can serve as an efficient tissue adhesive capable of holding the two skins together (**a**), normalized force-displacement curves for lap joints of two skins glued by different groups (**b**), neutralization of superoxide anions by MSN-Ceria in a dose-dependent manner in comparison with ceria aqueous suspension (**c**), and Scavenging activities of H_2_O_2_ by MSN-Ceria nanocomposites and ceria nanocrystals (**d**). (**B**) Representative images of the wound healing process during 8 days in vivo experiments (**a**), quantification of wound repair kinetics expressed as a percentage of the initial wound length (*n* = 5) (**b**), and microstructure of wound surfaces on day 22 after wound healing imaged using Nikon AZ100 stereomicroscope (**c**). *, *p* < 0.05. Reproduced with permission from ref [85]. Copyright 2018 Elsevier.

There is a growing demand for applying minimally invasive therapies, and the fabrication of effective adhesive materials is of great importance for enabling reconnection of surgical gaps and restoration of tissue integrity [115]. On this point, nanoparticles could act as adhesives for gels and biological tissues [116]. Specifically, the use of MSNs as tissue adhesive has been reported to be promising; Wu et al. could successfully attach ultra-fine nanoceria onto MSNs surface to form a potent functionalized ROS-responsive tissue-adhesive nanocomposite. This system showed admirable tissue-adhesive capacity in favor of fast wound closure plus lake of scar formation due to its capability of scavenging overproduced ROS at wound sites (see Figure 3) [85].

Generally speaking, prolonged inflammation is one of the main reasons for impaired wound healing due to the weakened protective role of the immune system and resultant bacterial infection [117]. This phenomenon originates from the low success rate of adhesives in chronic wounds [118,119]; for instance, glutaraldehyde cross-linked albumin adhesives could not be widely applied for pyogenic or granulomatous inflammation [120,121]. In this regard, inorganic nanomaterials exhibiting anti-inflammatory activity offer great opportunities in managing chronic skin wounds [122]. Loading anti-inflammatory drugs or other bioactive molecules into MSNs is likely beneficial for reducing inflammation responses and producing the next generation of adhesives.

### 5.4. MSNs in Skin Cancer Therapy

The significance of effective treatments for subcutaneous disorders comes from the fact that they are stated as the 4th leading cause of worldwide nonfatal diseases with timely diagnosis [123,124]. Extensive use of targeted MSN-based carriers in topical administration of anticancer drugs as a fascinating substitute for the systemic skin cancer treatment is due to the great affinity of MSNs for anti-neoplastic agents, which allows reducing the dose-related toxicity in order to overcome the restrictions of conventional chemotherapy [125,126]. In addition to the advantage of MSN-based carriers to moderate antitumor drug side effects due to indirect entering into the bloodstream, these nanomaterials can also increase drug penetration into the deep layers of the epidermis as well as significantly protect anticancer drugs against degradation. Based on preliminary in vitro studies, MSN carriers are the most innovative and promising strategy in topical anticancer applications. These nanomaterials were also incorporated in more complex drug delivery systems for the topical treatment of skin cancer, such as microneedle patches of photothermal indocyanine green-modified MSNs loaded with doxorubicin [127]. In addition, MSNs were successfully used for transdermal delivery of siRNA targeting TGF βR-1 (TGF βR-1) to the skin squamous cell carcinoma (SCC) in a mouse xenograft mode; the results showed a loading capacity of 1.4 μg of oligonucleotide per mg of MSNs and a 2-fold suppression of TGFβR-1 for MSNs containing TGFβR-1 siRNA as compared to controls [36].

As aforementioned, MSNs can act as powerful tools in the controlled release of pharmaceuticals (e.g., anticancer agents) at the destination point due to their attractive features, including long-term stability [128], extremely large surface area, as well as large pore volume leading to enhanced loading efficiencies [129]. As a point of fact, the silanol groups on the negatively charged surface of MSNs serve as adsorption sites for cargos with a positive charge, and it is feasible to load water-soluble therapeutic agents into MSN pores. Additionally, MBGs submicronic spheres are ideal candidates for cellular uptake by endocytosis thanks to their particle sizes range (100 to 300 nm).

**Figure 4:**
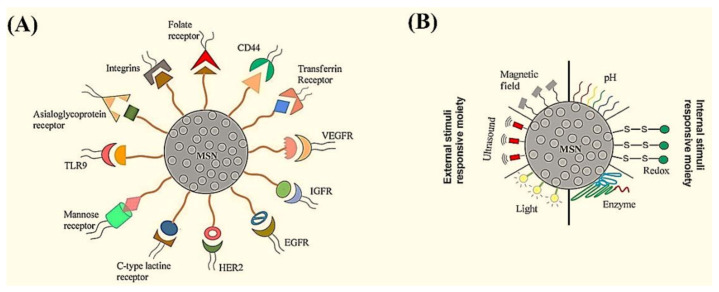
Schematic representation of (**A**) possible surface modifications of MSNs for active targeting to the receptors over-expressed in cancer microenvironment and (**B**) the main stimuli-responsive gatekeepers in decorated MSNs for controlled release of therapeutics in the tumor site. Reproduced from ref [130]. Copyright 2020 MDPI AG.

Several studies have previously confirmed the suitability of MSNs for targeted therapy of solid tumors; their surface could be easily tailored by varieties of ligands that target over-expressed receptors in the cancer microenvironment (Figure 4A) [130]. In 2015, targeted delivery of 5-aminolevulinic acid (5-ALA) for noninvasive photodynamic therapy of skin cancer was performed using folic acid-functionalized hollow MSNs; this system facilitated 5-ALA’s selective endocytosis bypassing through the lipophilic barrier to directly enter into B16F10 skin cancer cells. The in vitro results revealed high photocytotoxicity of the drug-loaded hollow MBGs to cancer cells upon red light irradiation for photodynamic therapy [131]. It has been highlighted that anticancer cargos may leak from mesopores of MSNs during the blood circulation and penetration into the tumor matrix, which results in insufficient drug concentration at the tumor site. In order to overcome this limitation, “smart” MSN-modified nano-systems with environment-responsive gatekeepers have been introduced. As regards the differences between tumor microenvironment and normal tissue characteristics (e.g., acidic pH, high concentration of glutathione, etc.), MSNs could be modified to represent the moiety sensitive to the tumor microenvironment and release the cargo specifically at the cancer site. Both internal (e.g., the pH, redox, and enzyme) and external (e.g., magnetic, light and ultrasound) stimuli-responsive gatekeepers can be applied to MSNs for providing controlled anticancer drug release (Figure 4B). For example, hybrid ultrasound- and temperature-sensitive copolymer-based MSN carriers were fabricated to deliver the anticancer drug 5-fluorouracil. The release of the drug molecules was controlled by adjusting the ultrasound frequency, which could also enhance drug permeation through the skin barrier [132].

As the combination of chemo-, photothermal and photodynamic therapies requires a high intensity of irradiation with superior anticancer ability, serious photo-toxicity to healthy neighboring cells may occur, which has limited its biomedical applications so far. Recently, an ultralow-intensity (0.25 W cm^−2^) NIR light nanoplatform has been developed to integrate chemo-/photothermal/photodynamic therapies upon 808 nm exposure below the maximum permissible skin exposure. The nanoplatform, consisting of a MSN shell co-loaded with the anticancer drug doxorubicin and chlorin e6 as photosensitizer (antitumor agent), could greatly promote drug release for improved chemotherapy. The in vitro and in vivo experimental results also revealed minimal photodamage with notable therapeutic efficiency [133].

In the search for suitable drugs, it was reported that amino acids mimicking nanotherapeutics could exhibit intrinsic anticancer targeting properties for skin cancer treatment. L-phenylalanine functionalized MSNs, for example, have achieved an overall suppression of tumor growth by 60% without the aid of any external stimuli or drugs [134]. Verteporfin-loaded MSNs could also inhibit the in vitro and in vivo proliferation of mouse melanoma by reducing the tumor mass of 50.2 ± 6.6% compared to the untreated (only glycerol) mice [135].

## 6. Mesoporous Bioactive Glasses (MBGs)

**Figure 5:**
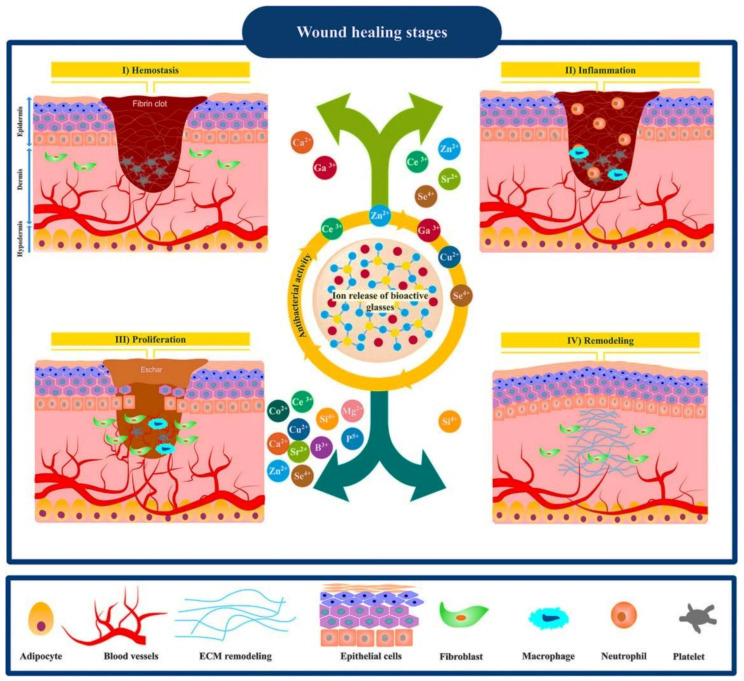
Schematic illustration showing four overlapping stages of the wound healing process (hemostasis, inflammation, proliferation, and remodeling) and potential effects of ionic dissolution products released from bioactive glasses (BGs) on each stage. Reproduced from [137]. Copyright 2020 American Chemical Society (ACS).

Mesoporous bioactive glasses (MBGs) combine the textural properties of ordered mesoporous silica along with the typical features of bioactive glasses. Their high specific surface area led to superior bioactivity, fast biodegradability, and quick release of bioactive ions. Moreover, their accessible pore volume allows the possibility of loading and releasing therapeutic biomolecules such as drugs and growth factors. Similar to MSNs, MBGs can also be prepared by a template-assisted sol–gel method involving an evaporation-induced self-assembly (EISA) process using surfactants or non-ionic block copolymers as structure-directing agents [136]. Usually, surfactants such as CTAB, P123, or FI27 are self-assembled into geometrical micelles or molecular aggregates, which act as templates for hydrolysis and polycondensation of MBG precursors. MBGs are synthesized in many forms, including particles, fibers, and scaffolds. Moreover, MBG particles can be incorporated into hydrogels, cements, ointments, and biopolymer scaffolds. The multifunctional merits of MBGs make them potential candidates for hard and soft tissue regeneration. However, MBGs are mainly explored in bone regeneration, while their application in wound healing and skin regeneration has only recently received growing interest. A set of properties should be exhibited by a bioactive material used for wound healing and skin regeneration since, basically, it should act as an antibacterial, anti-inflammatory, antioxidant, and angiogenesis stimulator. Interestingly, MBGs can be designed with all the required therapeutic properties through loading therapeutics (e.g., antibiotics, anti-and inflammatory drugs) into their mesopore volume along with the incorporation of therapeutic elements (e.g., Ag, Zn, Ce, Co) in their glass structure (Figure 5). Through careful design, therapeutics can be released in a short time or a long time, depending on their role in the regeneration process. For example, anti-inflammation therapeutics are required at the early stages of the regeneration process, whereas antibacterial therapeutics are required to be available for a longer time during the regeneration process.

## 7. MBGs for Wound Healing and Skin Regeneration

**Figure 6:**
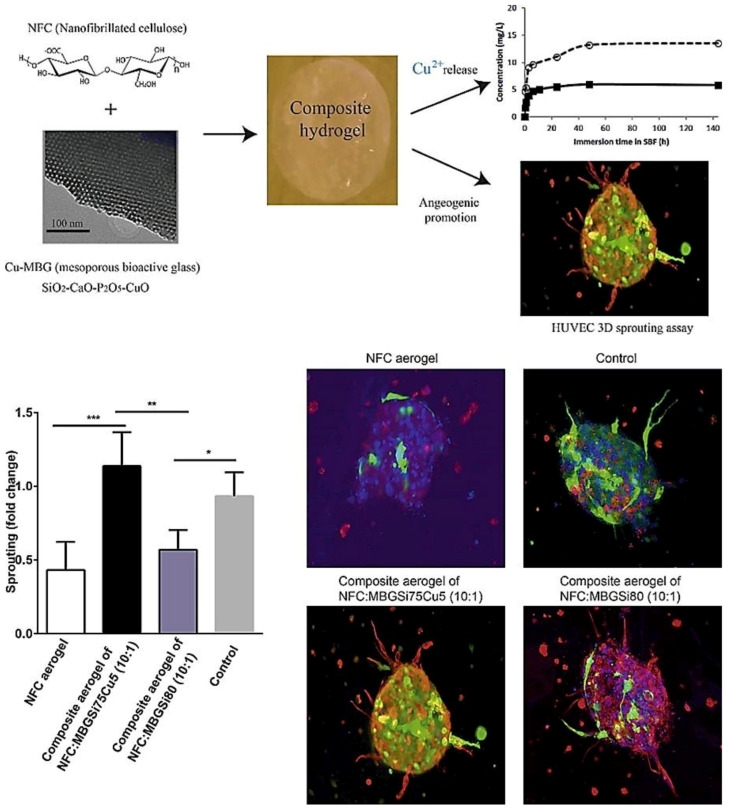
Sprouting of HUVEC spheroids with NFC, NFC: MBGSi80 (10:1) and NFC: MBGSi75Cu5 (10:1) composite aerogels: representative confocal images of expression of vimentin (in red) and fibronectin (in green) in HUVEC spheroids. The cell nuclei are counterstained with DAPI (blue). Bar = mean of sprout numbers ± s.e.m., *n* = 4. *—*p* < 0.1; **—*p* < 0.01; ***—*p* < 0.001. With permission from [138]. Copyright 2016 Elsevier.

The therapeutic multifunctionality of MBGs makes them highly interesting biomaterials to be applied in wound healing and skin tissue regeneration (Table 1). Acute wounds normally heal in a very orderly and efficient manner characterized by four distinct but overlapping phases: hemostasis, inflammation, proliferation, and remodeling (Figure 5). MBGs can be engineered to improve wound healing and skin regeneration through stimulating angiogenesis and inhibiting bacterial formation on the wound site. For example, Cu-containing MBGs were utilized in nanofibrillated cellulose matrix (NFC) composite aerogels to release Cu^2+^ ions for its angiogenic effect on promoting wound healing and its antibacterial effect as well (Figure 5) [138]. A 3D fibrin spheroid assay showed that NFC: MBGSi75Cu5 (10:1) aerogel significantly induced sprouting of human umbilical vein endothelial cells (HUVECs) and enhanced the expressions of vimentin and fibronectin in the HUVEC spheroid compared to control groups (Figure 6). This suggested that NFC: MBGSi75Cu5 (10:1) aerogel can promote fibroblast–endothelial cell interaction and ECM production, similar to the in vivo fibrin clot during the wound healing process.

## 8. MBGs for Skin Cancer Therapy

**Figure 7:**
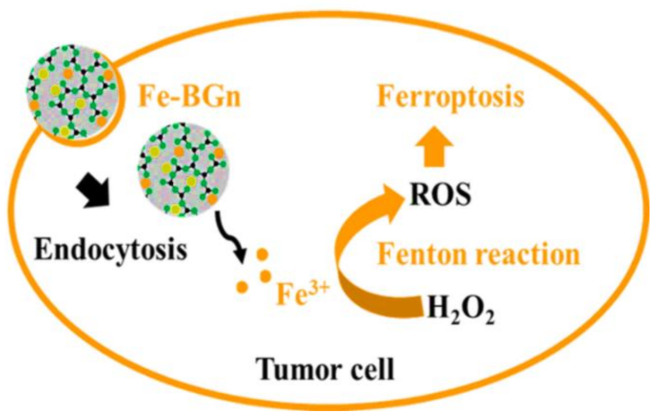
Application of Fe-MBG for ferroptosis-based cancer therapy. Reproduced with some modifications from ref [142]. Copyright 2021 Elsevier.

Cutaneous melanoma is the most aggressive form of skin cancer and one of the fastest-growing cancers worldwide, representing 60% of lethal skin tumors. MBGs can be used for targeted skin cancer therapy by acting as a local anti-cancer drug/ion delivery system. This kind of therapeutic strategy can potentially have high efficiency and excellent compliance to the patients. Interestingly, a new approach to using MBG in cancer therapy has recently been proposed by El-Fiqi and Kim [142]. This approach depends on using Fe-MBG for ferroptosis killing of cancer cells. Actually, ferroptosis is a kind of cell death resulting from accumulation of reactive oxygen species (ROS) such as the hydroxyl radical (•OH), which are generated through iron ion-mediated Fenton’s reaction (Fe^3+^ + H_2_O_2_ → Fe^2+^ + •OOH + H^+^ and Fe^2+^ + H_2_O_2_ → Fe^3+^ + •OH + HO^−^). Hydroxyl radicals (•OH) are powerful agents for rapid oxidation of membrane lipids as well as protein and DNA damage in tumor cells. Thus, Fe-MBG can kill cancer cells through the release of iron ions which would lead to intracellular Fenton’s reaction, producing ROS and ultimately induce tumor cell ferroptosis (Figure 7). Along with this, it has recently been reported that ferroptosis can suppress the growth of melanoma and may thus serve as a new treatment target for the research and development of drugs and/or treatment regimens [143]. Finally, ferroptosis-inducing drugs can also be loaded into Fe-MBG and provide synergism with the iron ions release.

### 8.1. MBGs for Hemostatic Applications

Hemostasis is one of the essential steps in skin repair. This process is an aggregate of cellular and biochemical activities that work together to preserve blood in the liquid state in the veins and arteries. Furthermore, it can impede blood loss after injuries through the formation of a blood clot. Up to now, a couple of methods and approaches have been developed to make hemostasis; the use of metallic elements (e.g., gallium and tantalum) are among the most promising strategies.

**Figure 8:**
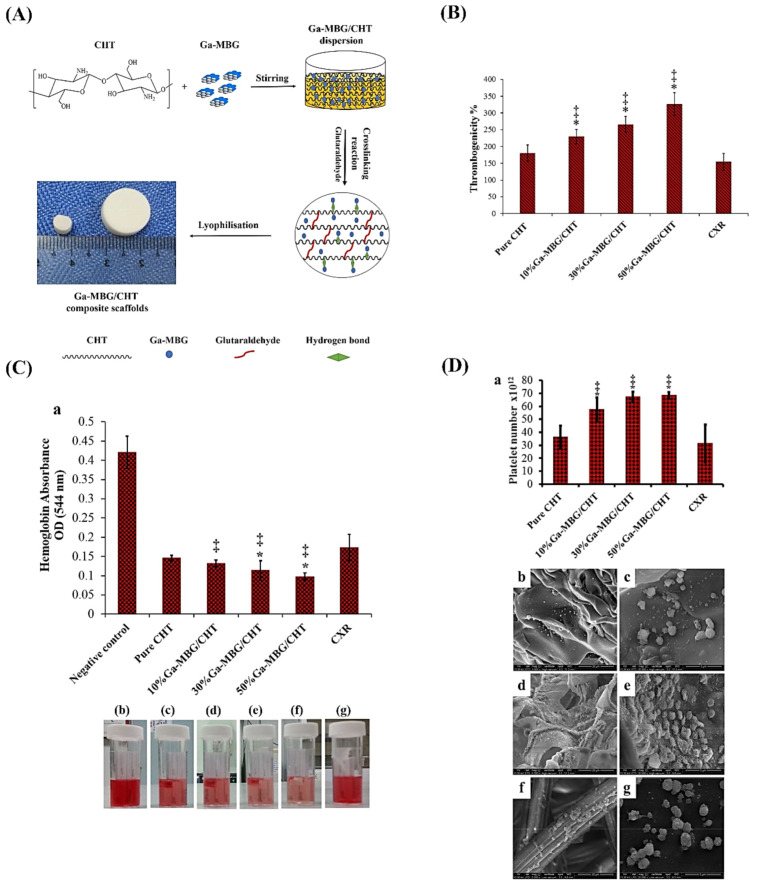
(**A**) Schematic illustration displaying the fabrication process of the Ga-MBG/chitosan composite scaffolds. The hydrogen bond between Ga-MBG and CHT may be formed via either the interaction of silanol groups of Ga-MBG with NH_2_ and OH groups of CHT or CHT with cationic ions of Ga-MBG (i.e., Ca^2+^ and Ga^3+^) through its functional groups. (**B**) Graph showing the effect of the composites on the thrombus formation over 30 min of incubation with whole blood. (**C**) Graph exhibiting blood clotting rate of composite scaffolds and CXR as determined by measuring hemoglobin absorbance (**a**) and photographs depicting more hemoglobin leaked from pure CHT, 10%GaMBG/CHT, and CXR than from 30%Ga-MBG/CHT and 50%Ga-MBG/CHT (**b**–**g**). (**D**) Effect of the scaffolds on platelet adhesion at 30 min post-incubation in PRP (**a**) and FESEM images of platelets adhered to the surfaces of pure CHT (**b**,**c**), 50%Ga MBG/CHT (**d**,**e**), and CXR (**f**,**g**). Note: * and ‡ demonstrate a significant difference compared with CHT and Celox^TM^ Rapid gauze (CXR) at *p* < 0.05, respectively. Reproduced from ref [139]. Copyright 2017 American Chemical Society (ACS).

Gallium, as an FDA-approved substance, is being used for treating a variety of skin wounds [144]. Gallium ions (Ga^3+^) showed the ability to induce blood clotting and platelet activation, making it a suitable additive in skin substitutes [145,146]. In addition to being hemostatic, Ga^3+^ ions show antibacterial activity against both Gram-positive (e.g., *Staphylococcus aureus* (*S. aureus*)) and Gram-negative (e.g., *Escherichia coli* (*E. coli*) and *Pseudomonas aeruginosa* (*P. aeruginosa*)) species found after skin lesions [147,148,149]. There are a couple of reports in the literature concerning Ga-doped MBGs developed for potential use in skin wound healing. As an illustration, Pourshahrestani et al. [150] reported a series of ordered MBGs ((80 − x)% SiO_2_ − 15% CaO − 5% P_2_O_5_ − xGa_2_O_3_ in mol%) doped with different contents of Ga_2_O_3_ (1, 2, and 3 mol%) and evaluated their hemostatic functions and antibacterial activity in vitro. Hemostatic activity of the samples was assessed by in vitro blood plasma coagulation evaluation (incubation time of 2 min), thrombus formation test (incubation time of 15, 30, and 60 min), and platelet adhesion (incubation time of 15, 30, and 60 min) assay. The antibacterial property of the glasses was evaluated by their incubation with E. coli and S. aureus at 37°C for 0, 1, 3, 6 and 12 h. The obtained results indicated that the inclusion of 1 mol.% Ga_2_O_3_ into MBG composition not only enhanced the biological properties (cytocompatibility, hemostatic performance, and antibacterial activity against both bacterial strains but also improved the structural properties (an increase of pore volume and the surface area near to 42% and 23%, respectively). The authors stated that the MBG samples containing the lowest concentration of Ga (1 mol.%) might be suitable hemostatic agents for controlling hemorrhage and infection. In a follow-up study, the same group has proposed Ga-doped MBGs for potential use in skin repair and regeneration [139]. To this end, they used the freeze-drying method for preparing composites made of chitosan and 1%Ga-MBG powder. The MBG formulation was 79SiO_2_–15CaO–5P_2_O_5_–1Ga_2_O_3_ (mol%) and synthesized by an evaporation-induced self-assembly (EISA) method. Three different concentrations (50, 30, and 10 wt%) of 1%Ga-MBG were added to the polymeric matrix to make composite scaffolds. Interestingly, the sample with the highest 1%Ga-MBG content (50 wt%) could increase thrombus generation, blood clotting, and platelet aggregation more than the hemostatic commercial product Celox^TM^ Rapid gauze (CXR) and pure CHT (see Figure 8). The cell viability assay indicated that the 1%Ga-MBG/CHT scaffolds have excellent compatibility with human dermal fibroblast cells (HDF). This may result from the fact that the incorporation of Ga-MBG into CHT matrix can improve its biological properties through increasing the surface roughness. Furthermore, the scaffolds containing 50 wt% of 1%Ga-MBG showed promising antimicrobial activity against skin bacteria including *E. coli* and *S. aureus*. Although these preliminary results seem somewhat promising, there are some limitations to the Ga-doped MBGs. For example, increasing the gallium content (>1%) in the glass structure may reduce the hemostatic performance of the final constructs. Furthermore, high concentrations of gallium (>1%) can be toxic to mammalian cells, suggesting caution in the use of this hemostatic agent [150].

Tantalum (Ta) has recently been recognized as another promising metal in managing soft tissue lesions [151]. In this regard, Mendonca et al. [140] synthesized Ta-containing MBGs ((80 − x)SiO_2_–15CaO–5P_2_O_5_–xTa_2_O_5_ (mol.%) by the EISA method. They then investigated the potential of this composition as a hemostatic agent by performing an in vivo animal study on mice (a tail-cut model). The results indicated that the MBGs containing higher amounts of Ta (>1%) show different physico-chemical properties in comparison with the Ta-free counterparts. For example, increasing Ta amounts (up to 10%) may result in a reduction of the pore volume and specific surface area of approximately 35% and 20%, respectively. Biologically, the samples containing Ta caused a substantial reduction in bleeding time (less than 50% of the average time) as compared with the bare MBGs and Arista^TM^, a commercial starch-based hemostat. Despite the promising results reported in recent articles, there is a paucity in experimental studies on Ta-doped MBGs for managing skin wounds; therefore, researchers and scientists of the field are suggested to design and develop such materials to reveal all pros and cons in vitro and in vivo.

### 8.2. MBGs for Antibacterial Applications

Chronic wounds, one of the most important clinical complications of surgery or pathological states like diabetes, are usually emphasized by some negative local factors such as bacterial infections that hinder the normal healing process of skin wounds [152]. The use of antibiotics seems the most common solution for preventing chronic wounds. However, bacteria can develop and become more resistant to the antibacterial activities of a wide range of antibiotics. For example, multidrug-resistant (MDR) phenotypes of *S. aureus* and *P. aeruginosa* have been identified, causing infectious wounds [153,154]. In this regard, the use of metallic antibacterial elements (e.g., Ag) is suggested as an appropriate alternative for inhibiting MDR bacteria [155,156,157,158,159]. As one of the most well-known antibacterial agents, Ag has been using for inhibiting and killing a broad range of bacterial species [160]. It has been well-documented that Ag ions (Ag^+^) exert their antibacterial effects via binding to bacteria DNA, RNA, and proteins, leading to the inhibition or killing of pathogens [161]. It should also be highlighted that Ag^+^ ions have more negative effects on Gram-negative bacteria as compared to Gram-positive species [162].

Previously, Ag and fluoride (F)-doped MBGs have been evaluated for antibacterial activity against MDR species [141]. The formulation of these sol–gel MBGs was as ([1 − (x + y)](58%SiO_2_ − 33%P_2_O_5_ − 9%CaO) − xCaF_2_ − yAg_2_O), where 0 ≤ x ≤ 20 and 0 ≤ y ≤ 2 mol.%. The incorporation of Ag_2_O and CaF_2_ into the MBGs altered the dissolution rate of the glasses. It was reported that 1% Ag-containing MBGs showed non-toxicity against fibroblasts (NIH 3T3 cells), while the 2% Ag-containing samples were toxic for the cells. Antibacterial activity of the glasses against *blaIMP* gene-positive *P. aeruginosa*, *Klebsiella pneumonia (K. pneumonia)*, *S. aureus*, and *E. coli* bacteria revealed that 1% and 2% Ag-doped MBGs inhibited the bacterial growth.

In another work, the research group of Prof. Boccaccini successfully developed MBG nanoparticles (96.60SiO_2_-3.40CaO mol.%) using a microemulsion-assisted sol–gel method for potential use in antibacterial applications. They used Ag for the surface modification of the samples and evaluated its effectiveness in the planktonic bacteria model and 3D infected skin model [52]. The morphology and porous shape of the particles remained unchanged after the modification, and the bioactivity of the glasses showed no decrease. Moreover, the particle size (100 to 250 nm) and pore sizes (2 to 9 nm) were not affected after the surface modification. Ag-modified MBG samples in the planktonic bacteria model showed a remarkable ability to inhibit *P. aeruginosa* and *S. aureus* compared to the pristine ones. Moreover, the 3D skin model revealed that the modified MBGs have only antibacterial activity against *S. aureus* (very close to the skin surface) while could not inhibit *P. aeruginosa* that attacks the deeper layer of the dermis. Although Ag-doped MBGs showed great promise for treating infectious wounds, the cytotoxicity of Ag^+^ ions is still the main limiting factor for huge usage of them in the clinic [163].

### 8.3. MBGs for Angiogenesis

Angiogenesis plays a critical role in successful wound healing since the formation of new granulation tissue requires new blood vessels [164]. Moreover, insufficient vascularization in the damaged sites may lead to the wound become chronic [164]. Therefore, the use of angiogenic substances seems an urgent need in managing skin wounds; in this regard, metallic elements such as copper exhibited an appropriate angiogenic potential [165]. Copper with different oxidation states, i.e., Cu^0^, Cu^+^, and Cu^2+^, showed a great ability to induce neo-vascularization [166], and its effectiveness for wound healing approaches has been well-documented [167]. It was showed that Cu^2+^ ions could trigger angiogenesis via the activation of the hypoxia-inducible factor-1 alpha (HIF-1α) and vascular endothelial growth factor (VEGF) [168]. Moreover, Cu^2+^ ions render antibacterial activity to different types of biomaterials (e.g., MBGs) [169,170]. Accordingly, several research groups over the globe have taken advantage of copper in various constructs for skin wound healing [171].

In order to examine the effectiveness of Cu-doped MBGs on skin wound healing, Wang et al. [138] prepared a series of composite wound dressings made of nanofibrillated cellulose (NFC) and in which MBGSi75Cu5 (5:1,1:1,10:1 and 5:2 NFC/MBG wt ratio) or MBGSi80 (10:1 NFC/MBG wt ratio) were added. The formulations of the glasses were MBGSi80 (Si, Ca, P = 80, 15, 5 mol%), MBGSi78Cu2 (Si, Cu, Ca, P = 78, 2, 15, 5 mol%), and MBGSi75Cu5 (Si, Cu, Ca, P = 75, 5, 15, 5 mol%). The obtained data clarified that all the Cu-doped MBGs possess two-dimensional hexagonally ordered mesopores in their structures, and the release of Cu from the samples happens in a controlled manner. In addition, the incorporation of Cu^2+^ into the glasses had no adverse effects on their in vitro bioactivity. The composite showed the possibility of suitably controlling the moisture on the wound. The in vitro viability assay revealed that amounts above 10 mg/L of copper might have inhibitory effects on the growth of 3T3 fibroblasts. The Cu-doped MBGs showed the ability to upregulate angiogenesis-related genes, including VEGFa, VEGFc, PDGFb, and FGF2 (bFGF) in the 3T3 fibroblasts. In a 3D spheroid culture model, the composite aerogels (NFC: MBGSi75Cu5 (10:1)) meaningfully induced the growth of human umbilical vein endothelial cells (HUVECs) and promoted the fibroblast–endothelial cell interaction and ECM production. In addition to angiogenesis, some of the prepared composites (NFC: MBGSi75Cu5 = 5:2 and NFC: MBGSi75Cu5 = 10:1) showed the capability of preventing the growth of *E. coli*.

In another study, Paterson et al. [40] evaluated the angiogenic and antibacterial activities of Cu-doped and undoped silicate MBGs with the compositions of 85SiO_2_/13CaO/2CuO mol% and 85SiO_2_/15CaO (mol%). The prepared samples showed a very high specific surface area (740 m^2^/g) and a uniform pore (size 4 nm) in their structure. The release of Cu^2+^ ions from the glasses was recorded over 14 days, while the highest release amounts took place in the first 72 h. The concentration of 30 and 300 μg/mL of the Cu-doped MBGs enhanced the growth of endothelial cells (ECs) in vitro. The authors demonstrated a noteworthy antibacterial potential of the sample against both planktonic and biofilm bacteria. Unlike the comparative commercial dressing (Acticoat Flex3^TM^), the samples showed no cytotoxicity in either two-dimensional cell monolayers or a 3D human skin model. Regarding the literature, it should be pointed out that, although copper may be useful in skin wound healing, its local toxicity and the risk of accumulation are the main concerns for in vivo and clinical studies.

## 9. Conclusions and Future Challenges

A lot of experimental studies carried out over the last decade have convincingly proved that mesoporous silicate biomaterials—MSNs and MBGs—are suitable for applications not only in contact with bone but also in the fields of wound management and skin tissue engineering. Of course, there is an obvious mismatch between the physico-mechanical properties of these hard and rigid inorganic materials and those of soft tissues; hence, in most cases, they need to be embedded in polymeric matrices to obtain softer composites. This approach also carries the advantage of making the topical application of these materials particularly easy, which is so common for treating skin or superficial injuries.

MSNs MBGs exhibit an exceptional conformational versatility as they can be produced according to a variety of structures and morphologies (e.g., external geometry, mesopore size, and, in general, textural properties): therefore, a “universal” criterion of choice cannot be defined, but the type, shape, size, and dosage of these materials should be carefully selected depending on the specific application.

MSNs and MBGs have in common the property of acting as drug delivery vehicles: textural characteristics (primarily mesopore size) can be properly tailored depending on the synthesis process, the parameters of which can be selected on the basis of the specific biomolecule to be hosted. As compared to MSNs, MBGs have the additional extra-functionality of acting as vehicles for the release of therapeutic ions as well. In principle, the synergy between the release of drugs/growth factors from the mesopores and ions upon material dissolution can lead to potentiated effects and better therapy; however, the interactions between these two agents, including mutual inhibitory and side effects, should be carefully evaluated. This is a highly fascinating field of research, but, at present, there is a lack of specific regulations and protocols, also in terms of ad hoc in vitro and in vivo models, to study such synergistic interactions.

The release of ions and biomolecules can be finely modulated if the walls of mesopores are properly functionalized and/or the mesoporous materials are embedded in stimuli-responsive polymeric matrices: in both cases, mesopores behave as “intelligent gates” that can be selectively opened or closed depending on the conditions of the environment (e.g., bloodstream, intact surface of the skin, injured skin/wound, etc.) These smart biomaterials also carry the added value of minimizing toxicity in non-target tissues, allowing localization of the release and associated therapeutic actions only when the carrier reaches the wound region, which may exhibit peculiar biochemical conditions, e.g., pH, that are different as compared to those of intact skin. Indeed, a wise, safe, and reproducible exploitation of this therapeutic approach requires the understanding and selection of the most suitable biochemical stimuli that can activate such intelligent MSN-/MBG-based systems.

## Figures and Tables

**Table 1:** A short list of experimental studies performed on ion-doped MBGs for potential use in wound healing.

Composition	Synthesis Method	Dopant	Application	Remarks	Ref
1%Ga-MBG (79SiO_2_–15CaO–5P_2_O_5_–1Ga_2_O_3_) (10, 30, and 50 wt%) with CHT	Sol–gel using EISA with freeze-drying	Ga	Hemostatic and antibacterial	- 1% Ga-MBG content can increase blood clotting and platelet aggregation compared with pure CHT and CXR. - The cell viability confirmed exceptional biocompatibility of Ga-MBG/CHT composite scaffolds in contact with HDF cells	[139]
(80 − x) SiO_2_ − 15CaO − 5P_2_O_5_ − xTa_2_O_5_, where x = 0, 0.5, 1, 5, 10	Sol–gel using EISA	Ta	Hemostatic	- A substantial reduction in bleeding time (more than 50% of the average bleeding time) was observed for Ta-MBGs compared to Arista^TM^, and MBGs without Ta	[140]
([1 − (x + y)] (58SiO_2_ − 33P_2_O_5_ − 9CaO) − xCaF_2_ − yAg_2_O), where 0 ≤ x ≤ 20, 0 ≤ y ≤ 2	Sol–gel	Ag	Antibacterial	- Antibacterial properties in 1% silver-containing BGs of mass <1 mg/mL against *blaIMP* gene-positive *P. aeruginosa, K. pneumonia, S. aureus,* and *E. coli* bacteria	[141]
MBGN (96.60SiO_2_-3.40CaO) and MBGN with Ag	Sol–gel using EA-CTAB-water micro-emulsion droplets	Ag	Antibacterial	- Enhanced antibacterial activity in samples surface-modified Ag-doped MBGs against *P. aeruginosa* and *S. aureus* at a concentration of 1 mg/mL - No sign of cytotoxicity against fibroblasts in vitro	[52]
MBGSi80 (molar ratio Si/Ca/P = 80/15/5)&&&MBGSi78Cu_2_ (molar ratio Si/Cu/Ca/P = 78/2/15/5) and MBGSi75Cu_5_ (molar ratio Si/Cu/Ca/P = 75/5/15/5)	Sol–gel using EISA	Cu	Angiogenic and antibacterial	- The aerogel NFC: MBGSi75Cu5 (10:1) showed angiogenic activity at biological levels (<10 mg/L)	[138]
85SiO_2_–13CaO–2CuO	Ultra-sound-assisted base catalyzed sol–gel method	Cu	Angiogenic and antibacterial	- The proangiogenic effect increases and outgrowths ECs at a concentration range between 30 and 300 μg/mL raising - Cu-MBG at 100μg/mL shows antibacterial effects against *P. aeruginosa* and *S. aureus*	[40]

Abbreviations: CHT, chitosan; CXR, Celox^TM^ Rapid gauze; EISA, evaporation induced self-assembly; HDF, human dermal fibroblast; MBGs, mesoporous bioactive glasses; NFC, nanofibrillated cellulose.

## Data Availability

No new data were created in this study.
